# No Sting in the Tail for Sterile Bisex Queensland Fruit Fly (*Bactrocera tryoni* Froggatt) Release Programs

**DOI:** 10.3390/insects13030269

**Published:** 2022-03-09

**Authors:** Olivia L. Reynolds, Damian Collins, Bernard C. Dominiak, Terry Osborne

**Affiliations:** 1 New South Wales Department of Primary Industries, Biosecurity and Food Safety, Elizabeth Macarthur Agricultural Institute, Private Bag 4008, Sydney, NSW 2567, Australia; damian.collins@dpi.nsw.gov.au (D.C.); terry.osborne@dpi.nsw.gov.au (T.O.); 2 Graham Centre for Agricultural Innovation, Charles Sturt University and New South Wales Department of Primary Industries, Locked Bag 588, Wagga Wagga, NSW 2678, Australia; 3 Susentom, Heidelberg Heights, Melbourne, VIC 3081, Australia; 4 New South Wales Department of Primary Industries, The Ian Armstrong Building, 105 Prince Street, Orange, NSW 2800, Australia; bernie.dominiak@dpi.nsw.gov.au

**Keywords:** management, control, trade, markets, female, *Helicoverpa*, biosecurity, peach, plum, nectarine

## Abstract

**Simple Summary:**

Fruit fly (Tephritidae) present a global market-access issue for horticultural produce. A key method of control for tephritidae pests is the sterile insect technique (SIT). Australia has released a bisex strain, i.e., males and females of sterile Queensland fruit fly, *Bactrocera tryoni* Froggatt, although only the males contribute to wild-population decline. While the number of stings were higher in one SIT release orchard, compared with the control, we showed that sterile female *B. tryoni* released in large numbers do not lead to degraded or unmarketable fruit, and therefore are suitable for release in commercial nectarine, peach and plum orchards.

**Abstract:**

Global markets do not tolerate the presence of fruit fly (Tephritidae) in horticultural produce. A key method of control for tephritidae pests, is the sterile insect technique (SIT). Several countries release a bisex strain, i.e., males and females, however the sterile male is the only sex which contributes to wild population declines when released *en masse*. In commercial orchards, there are concerns that sterile females released as part of bisex strains, may oviposit, i.e., ‘sting’ and cause damage to fruit, rendering it unmarketable. Australia has released a bisex strain of sterile Queensland fruit fly, *Bactrocera tryoni* Froggatt, for several decades to suppress wild pest populations, particularly in peri-urban and urban environments. Here, we assessed fruit damage in two commercially grown stone fruit orchards where bisex sterile *B. tryoni* were released, and in an orchard that did not receive sterile flies. The number of detected stings were higher in only one SIT release orchard, compared with the control; however, there was no difference between SIT and control orchards in the number of larvae detected. We showed that there is no evidence that sterile female *B. tryoni* released in large numbers caused stings, or damage that led to downgraded or unsaleable fruit. The bisex strain of sterile *B. tryoni* is recommended for use in commercial stone-fruit orchards, under the conditions in which this trial was conducted.

## 1. Introduction

Globally, fresh fruit markets have nil tolerance for fruit fly (Tephritidae) damaged fruit. The sterile insect technique (SIT) is a form of biological control whereby large numbers of mass-produced insects are irradiated to render them sterile. These sterile insects are transported to release centres [1,2], and then released into the target area to flood the wild pest population, reducing the possibility of mating occurring between wild females and wild males and the subsequent production of fertile eggs [3]. Some SIT programs, such as for the Mediterranean fruit fly (*Ceratitis capitita* Wied.), have developed genetic sexing strains which facilitates the ready separation of the sexes so that only sterile males are released [4], the only sex which effectively contributes to SIT. While the use of bisex strains, i.e., male and female strains, as part of SIT programs was largely discontinued for *C. capitata* in the early 2000’s [5], other species may still be released as bisex strains, despite little published evidence of whether sterile females sting fruit, and whether these stings lead to the unmarketability of the fruit. Grower confidence in Area Wide Management (AWM) programs that incorporate SIT could be improved if it can be shown that sterile females do not cause damage to mature fruit. This confidence would likely result in increased grower-willingness to invest in AWM and SIT [6].

The Queensland fruit fly, *Bactrocera tryoni* (Froggatt) (Qfly) is considered Australia’s most severe horticultural pest due to the direct damage it causes to numerous horticultural crops [7]. Adult female *B. tryoni* oviposit into ripening and mature fruit and the developing larvae feed on the flesh of the fruit, thereby rendering it inedible and unsuitable for sale. There are many factors that influence egg laying in different fruits (see [7] for a summary). Qfly frequently prefer to oviposit into breached fruit skin, apparently because the skin does not have to be penetrated with their blunt ovipositor. Fruit skin can be damaged by many factors such as other insects including *Helicoverpa* spp. Therefore, insect control is a key component of orchard management to minimize Qfly infestation.

Many importing countries, states and territories are sensitive to potential *B. tryoni* incursion and impose significant domestic and international market restrictions for fruit and vegetables [8]. In 2016–2017, the produce potentially affected by fruit flies was valued at $6 billion, which is about half of the value of the Australia’s horticulture sector of $12 billion/annum [9].

Chemical products traditionally used in pre-harvest fruit production have now been withdrawn from sale or their use is greatly restricted in Australia [8,10]. Therefore, there has been an increased interest in SIT as a tool to manage *B. tryoni*, particularly for several horticultural production areas in the south and south eastern states of Australia. Conversely, SIT has been used in urban and peri-urban situations bordering horticultural growing regions for several decades to reduce the pressure on horticultural regions [11], and to manage outbreaks in areas the pest is not endemic [12,13].

In Australia, a bisex strain of *B. tryoni* are released, despite only the sterile males contributing to the control of wild populations [14]. The development of a viable single sex strain of *B. tryoni* for use in SIT programs remains elusive. Despite the long-term urban and peri-urban use of a bisex *B. tryoni* strain in Australia, there remains concerns that sterile females may oviposit, i.e., ‘sting’ and cause damage to fruit, rendering it unmarketable. In urban situations, stinging fruit is usually not a concern because backyard fruit is frequently poorly managed [15], grown opportunistically and not for profit.

Our aim was to determine the level of oviposition or attempted oviposition by sterile and wild female flies on mature fruit by examining commercially grown fruit in a packing shed, and if this damage led to any impact on the market access of the fruit across several orchards.

## 2. Materials and Methods

### 2.1. Study Area

The study area was located in the Southern Downs region of Queensland, which has a strong horticultural industry with 4210 hectares of production by 277 horticultural producers with a value of nearly $300 million at the wholesale level [16]. There are 1446 hectares of orchard crops, mainly apples, followed by stone fruit, with several small areas of pears, persimmons, figs and olives [16].

The participating commercial orchards in the region included Warroo (28°36′07.32″ S, 151°26′21.59″ E; 60 Ha), Traprock (28°44′09.91″ S, 151°31′23.44″ E; 35 Ha) and Top Lawson (28°40′24.69″ S, 151°31′46.47″ E; 15 Ha), collectively growing a total of approximately 110 Ha of medium chill stone fruit. Pikes Creek packed the fruit from Warroo and Top Lawson orchards, while Traprock packed their own fruit. These orchards are geographically isolated from urban centres, and aside from the orchards, the surrounding area is largely grazing land. The stone fruit across the three orchards is harvested from late October through to late February depending upon the variety. Warroo only grows plums (Queen Garnet) while Traprock and Top Lawson orchards all have a mix of plums, peaches and nectarines. Traditionally, the orchards (except Warroo, which was newly planted in 2012) have followed a conventional program to control fruit flies which relied principally upon insecticide (largely fenthion prior to withdrawal of its use in Australia) with the sporadic use of the male annihilation technique and other controls. Sanitation involved the collection and destruction of discarded fruit. In the present study, Warroo and Traprock used sterile flies as part of their fruit fly management program (SIT), while Top Lawson was the control orchard and did not release sterile flies (C).

### 2.2. Sterile Insect Releases

*Bactrocera tryoni* pupae (bisex strain) were obtained from the Fruit Fly Production Facility (FFPF) at the Elizabeth Macarthur Agricultural Institute, Menangle, New South Wales (NSW), Australia (see [17] for details of the processes). Pupae were dyed with a fluorescent pigment (Fiesta FEX 1 Magenta, Swada, 30–32 Kilkenny Court, Dandenong South, Victoria, Australia) by inverting the dye and pupae in a plastic bag to evenly coat the pupae. The standard pigment and rate used to mark sterile *B. tryoni* for SIT programs of 0.8 g dye per 100 g pupae in Australia was followed [18,19]. The dyed *B. tryoni* were placed in anoxic conditions [20] and sent to the Australian Nuclear Science and Technology Organisation (ANSTO), Lucas Heights, New South Wales where they were irradiated at 60.0–65.0 Gy to render them sterile [21].

### 2.3. Transport

At the start of each week, pupae were packaged in cardboard boxes on a Monday, irradiated at ANSTO on a Tuesday and transported in polystyrene boxes by air (Sydney to Brisbane) and road (Brisbane to Southern Downs) to arrive at Traprock orchards on a Thursday, and Warroo orchard on a Friday. Upon receipt, the plastic bags containing the pupae were opened and distributed into the release containers (up to 25,000 pupae/release container). Temperature during transit ranged from 17.7–25.8 °C and relative humidity ranged from 47–83%.

### 2.4. Release Protocol

This trial released the bisex strain of *B. tryoni*, i.e., sterile males and females at a ratio of about 50:50 [17]. As the commercial orchards were under an AWM SIT trial program, of which assessing sterile female stings was only one component, we were conservative with the number of sterile males released. The mean number of released sterile male *B. tryoni* was calculated using the number of pupae sent to the orchards each week (based on the mean weight of pupae for each year) and the mean number of fliers pre-transport, assessed at the FFPF following the standard protocols [17].

The *B. tryoni* bisex strain were released as adults, by growers who were trained by the project team, following the methods detailed in Reynolds and Orchard [22]. Briefly, the pupae were placed in well-ventilated boxes, acclimated under local conditions and reared to adult under a roofed farm shed at the release site [16], and released when the majority were aged 2–3 days. All releases were terrestrial. A full diet (yeast hydrolysate, sugar and water) to promote maturity and longevity was offered at eclosion [19]. Releases commenced in September 2016 and continued weekly until their cessation in March 2017.

### 2.5. Sterile Fruit Fly Female Stings and Helicoverpa *spp.* Larvae Damage

During the 2016/2017 harvest, fruit from the three orchards was examined at the packing sheds located at Traprock and Pikes Creek orchards, Stanthorpe Qld. The same individual conducted all the inspections, with an additional person assisting with the Warroo orchard inspections. The dates of harvest and fruit (including cultivars) are provided in Supplementary Table S1. Fruit was examined for *B. tryoni* stings, eggs, larvae and any damage caused by *Helicoverpa* spp., and recorded. Fruit was randomly selected from the start of the packing line prior to inspection and sorting by staff in the packing shed. To inspect for stings, each piece of fruit was wiped with a paper towel to remove any white gloss and examined with the aid of a LED Headband Magnifier using the 1.5× magnification setting. The fruit was examined from the top around the stalk and rotated to examine the sides and the base. When a sting was found, a scalpel blade was used to cut open the fruit and the sting was washed through a fine sieve into a water bath with a black filter paper base so that any eggs would be visible. The number of fruits counted on each day was 2% of the day’s pick or 600 individual whole fruit (whichever was greater) [23]. The variety of each whole fruit was recorded along with the orchard name. Damage was recorded for Lepidoptera (*Helicoverpa* spp.), at each orchard, if larval boring holes were evident.

### 2.6. Statistical Analysis

The presence of at least one sting, or *Helicoverpa* spp. damage (score > 0), were modelled as binary in a generalized linear mixed model consisting of the fixed effects of orchard, fruit type and variety (within fruit type), and random effects of date and bin (within date) (for stings, date by fruit type and date by bin by fruit type were added) in the ASReml-R package [24] of the R statistical environment [25]. Replicate means for larvae were analysed using an ANOVA, as larval numbers were limited. We used a chi squared test of association on the two-way contingency table to examine the association between the presence of *Helicoverpa* spp. and fruit fly stings. Firstly, for all the data and secondly by date (to confirm the association or lack thereof). Further, the % prevalence of stings was calculated for where *Helicoverpa* spp. was present versus where *Helicoverpa* spp. was absent to aid in the interpretation of the association (or lack thereof).

The comparisons between orchards were confounded with time of year (since each orchard was sampled on a different set of days) and varieties (since different plum varieties were assessed at each orchard). In addition, Nectarine was only sampled on one day. Comparisons between fruits (for Traprock) were somewhat confounded with day—for instance, the White Peach (WP) were sampled on the first 3 days, whereas Plum was sampled on the last 2 days.

## 3. Results

### 3.1. Sterile Flies

On average, the effective sterile male fliers for 2016/2017 were 3279 males per Ha/week. Any impact of environmental conditions at the orchard sites, on emergence and release were not factored into these values. These means include the increased targeted releases of sterile *B. tryoni* that occurred in hot spots as required and indicated by trapping.

### 3.2. Fruit Assessment

Over 19,000 whole fruit from three orchards (Top Lawson (C), Traprock (SIT), and Warroo (SIT)) were examined for *B. tryoni* stings, eggs, larvae as well as any *Helicoverpa* spp. damage (no *Helicoverpa* spp. damage was detected at Traprock). No *B. tryoni* eggs were found in any of the examined fruit. Table 1 provides information on larval detections.

### 3.3. Fruit Fly Stings

For the presence of stings (>1 sting), there were significant effects of orchard (F_2,12_ = 24.1, *p* < 0.001; Table 1). Traprock orchard (SIT) and Top Lawson (control) did not differ in the mean number of ovipositor excavations or stings. However, Warroo orchard (SIT) had a significantly greater number of stings per fruit than Top Lawson (control), although not significantly different to the other SIT orchard (Traprock). There was a significant effect of fruit type (within Traprock) (F_3,15_ = 13.23, *p* < 0.001) (Table 2), but no significant effects of variety (within fruit and orchard) (F_3,25_ = 0.37, *p* = 0.776), on the number of observed stings. Of the variance components, date by fruit and date by bin were non-zero but very small (in comparison to the residual (binary) variation).

For plum, the predicted proportion of fruit with stings was highest for Warroo (7.7%) (SIT), then Traprock (5.1%) (SIT), then Top Lawson (1.8%) (C)). For Traprock (SIT), the proportion of stings was highest in Nectarine (9%), followed by Plum (5.1%), then Yellow Peach (2.3%) and White Peach (0.9%) (Table 2).

### 3.4. Fruit Fly Larvae

There was no significant difference in the number of larvae per fruit between the orchard treatments (F_2,3_ = 0.656, *p* = 0.589; Table 1).

### 3.5. Helicoverpa *spp.* Damage

For the presence of *Helicoverpa* spp. damage (score > 0), there was a significant difference between the two orchards (F_1,6.7_ = 32.6, *p* < 0.001) but no difference between plum varieties (F_1,7_ = 2.21, *p* = 0.181). The proportion of fruit with *Helicoverpa* spp. damage was higher in Warroo (11.2%) (SIT) than Top Lawson (1.2%) (C) (Table 3).

### 3.6. Fruit Fly Stings vs. Helicoverpa *spp.*

Overall, there was no indication of an association between *Helicoverpa* spp. and fruit fly stings (X^2^_1_ = 1.61, *p* = 0.204). The proportion of stings was similar where *Helicoverpa* spp. damage was present (6.65%) and where *Helicoverpa* spp. damage was absent (5.65%) (Table 4 and Table 5).

## 4. Discussion

Fruit fly stings were detected throughout the duration of the trial, in both control and treatment orchards, however, they were higher (although only significant for one) in orchards that received sterile flies, as may be expected; however there was no difference between SIT and control orchards in the number of larvae detected. We suspect that the detected fruit fly stings, where there was no evidence of eggs, larval development or feeding, were likely aborted stings or merely excavations. The stings (aborted or otherwise) were probably caused by either sterile or wild females that were unmated, or had mated with sterile males. While stings were evident to the trained eye of entomologists, there was no issue with the sale of stone fruit for the entire season due to fruit fly (Rowan Berecry pers. comm. 18 April 2017). Thus, there were no concerns about the impact of sterile female fruit fly stings on the market access of stone fruit.

Inspections of mature plums showed a higher number of stings (but not larval infestation) in those orchards that released sterile flies, compared with the control orchard that followed standard management practices. Fruit fly stings are typically not visible except to a trained eye, unless feeding damage is evident. In our study, ineffective stinging (i.e., nil presence of eggs and/or larvae) did not cause any discernible damage to the inspected fruit. Indeed, during the 2017 packing of SIT treated Warroo Orchard plums, it was observed that “To date we have packed 170,000 kg of Queen Garnet and have not seen any damage to fruit that could be attributed to sting marks caused by sterile Queensland fruit fly” (Andy Finlay pers. comm., Chair Summer Fruit Australia, 7 February 2017). Field-grown stone fruit is rarely unblemished, and may experience a range of imperfections, both minor, and more severe such as sunburn, hail damage, lepidopteran larval feeding and thrips damage.

There is no technique that can differentiate between wild and sterile female fruit fly stings, nor indeed between the stings caused by different fruit fly species. As the control orchard, i.e., no sterile flies released, also recorded stings, we suggest that abortive stings by wild females are likely to have contributed to up to nearly half of fruit fly stings in the SIT treated orchards.

Several authors mention sterile fly stinging of fruit [4,26,27], however, it is not clear if this is a perceived threat or a documented problem. Irrespectively, many fruit fly programs have adopted the male only strain at a considerable expense. However, there are a range of pros and cons to the choice of either a bisex or male-only strain, in addition to cost, for example the target area (urban, peri-urban or commercial farm), resources and ability to develop a male-only strain and fly performance [4,26,27], which may lead to the decision to choose one over the other.

Different host fruit are known to differ in their ability to support the fruit fly life cycle [28,29]. The gradation from least frequently stung to most frequently stung (SIT) was white peach, yellow peach, plum and nectarine. This follows anecdotal evidence that suggests that female fruit fly are deterred from oviposition, or have a reduced preference for oviposition in peaches due to the ‘fuzzy’ coating, compared to smoother skinned stone fruit, while there is no official ranking of *B. tryoni* host plants, certain fruit types, for example, stone fruit (particularly apricots), mangoes, tomatoes (most of which are grown commercially under protected cropping conditions in which fruit flies are not typically a problem). Furthermore, guava and feijoa are known to be particularly favourable to Qfly [29], while others including citrus are considered less favourable [30]. There are several studies that have ranked different cultivars, for example a host susceptibility index was used to rank six citrus cultivars and revealed, in order of most susceptible to least susceptible, Murcott mandarins > Imperial mandarins ≥ Navel oranges ≥ Ellendale mandarins > Valencia oranges ≥ Eureka lemons (yellow) > Eureka lemons (green) [30]. Further research is warranted across a broad range of fruit types to identify the functional traits of host fruits that are important for successful oviposition and for the successful completion of development.

There was little difference in the proportion of fruit fly stings in the presence or absence of *Helicoverpa* spp. Fruit fly stings did not increase in orchards where *Helicoverpa* spp. are present, at least at the levels observed in this study. This could be a benefit for orchards utilising organic control methods, low input systems, or where fruit is grown for pesticide-sensitive markets.

## 5. Conclusions

Our study found no evidence that sterile female *B. tryoni* released in large numbers caused stings that lead to downgraded or unsaleable fruit. Furthermore, the release of a bisex *B. tryoni* strain caused no market access issues in stone fruit. Our studies support a recommendation for the use of the current *B. tryoni* bisex strain in AW-IPM SIT programs.

## Figures and Tables

**Table 1:** Fruit fly stings and larvae recorded from stone fruit sampled at Pikes Creek packing shed from sterile and control orchards during the 2016/2017 stone fruit season.

Orchards	Total Fruit Sampled	Total Stings	Total Larvae	Mean Number of Fruit Fly Stings Per Fruit	Mean Number of Fruit Fly Larvae Per Fruit
Top Lawson (control)	4674	128	10	0.027 a	0.002 a
Traprock (SIT)	6807	442	99	0.065 ab	0.015 a
Warroo (SIT)	8315	902	21	0.109 b	0.003 a

Within each column, values followed by the same letter are not significantly different from one another (*p* > 0.05).

**Table 2:** Fruit type and orchard effects on proportion of *B. tryoni* stings (Mean ± SE from the sting analysis (logistic scale) and predicted proportion of *B. tryoni* stings ± approx SE (back transformed).

	Top Lawson (C)	Traprock (SIT)	Warroo (SIT)
Mean number of fruit with at least one sting			
Nectarine		−2.32 ± 0.30 c	
Plum	−4.02 ± 0.18 A	−2.92 ± 0.25 Bc	−2.49 ± 0.12 B
White Peach		−4.73 ± 0.29a	
Yellow Peach		−3.75 ± 0.18b	
Predicted proportion of stings ± approx SE			
Nectarine		0.090 ± 0.0219 c	
Plum	0.018 ± 0.0029 A	0.051 ± 0.0110 Bc	0.077 ± 0.0079 B
White Peach		0.009 ± 0.0022 a	
Yellow Peach		0.023 ± 0.0036 b	

Within each column, values followed by the same uppercase letter are not significantly different from one another (*p* > 0.05), and similarly for fruit with the same lowercase letter.

**Table 3:** Predicted proportion of fruit sampled with *Helicoverpa* spp. damage (score > 0) by fruit type and orchard, showing the mean ± SE on the logistic scale and predicted proportion ± approximate SE (back-transformed).

Orchard	Mean ± SE	Proportion
Top Lawson (C)	−4.44 ± 0.57	0.012 ± 0.010
Warroo (SIT)	−2.08 ± 0.66	0.112 ± 0.050

**Table 4:** The total number of fruit with and without fruit fly stings with recorded *Helicoverpa* spp. damage across Warroo and Traprock orchards.

*Helicoverpa* spp. Damage	Fruit with No Fruit Fly Stings	Fruit with Fruit Fly Stings	Percentage Fruit Fly Stings (%)
*Helicoverpa* spp. damage evident	11262	674	5.65
*Helicoverpa* spp. damage not evident	983	70	6.65

**Table 5:** *Bactrocera tryoni* stings and *Helicoverpa* spp. damage across all sampling dates. H− = *Helicoverpa* nil damage, H+ = *Helicoverpa* damage, S− = Nil fruit fly sting(s), S+ = Sting(s) observed.

Date	Number of Fruit with and without Damage from Fruit Fly or *Helicoverpa* spp.				Chisq Stat	*p*-Value	Percentage of Fruit with Fruit Fly Stings	
	H− S−	H− S+	H+ S−	H+ S+			H−	H+
20 December 2016	752	10	17	1	0.248	0.619	1.3	5.6
21 December 2016	588	6	0	0	-	-	1.0	-
22 December 2016	1908	45	25	2	1.2	0.274	2.3	7.4
23 December 2016	1282	26	12	0	0	1.000	2.0	0
30 December 2017	1199	122	52	7	0.203	0.653	9.2	11.9
31 January 2017	1276	143	172	19	0	1.000	10.1	9.9
1 February 2017	1061	112	108	14	0.274	0.601	9.5	11.5
7 February 2017	1180	70	209	11	0.0398	0.842	5.6	5.0
8 February 2017	1171	77	213	9	1.17	0.279	6.2	4.1
9 February 2017	845	63	175	7	1.93	0.165	6.9	3.8

## Data Availability

Not applicable.

## Supplementary Materials

The following are available online at https://www.mdpi.com/article/10.3390/insects13030269/s1, Table S1: Mean number of stings and *Helicoverpa* spp. damage across orchards and dates of sampling: number of bins, average fruit/bin sampled, and fruit type (Pl = Plum, WP = White Peach, YP = Yellow Peach, Nc = Nectarine). and variety (QG:Queen Garnet, SA:Snow Angel, MP:May Princess, SP:Spring Princess, ZF:Zee Fire, BN:Black Nectar, CL:Crimson Lady, ET:Ebony Treat, PM:Purple Majesty).
