# Supplementary material for: No Sting in the Tail for Sterile Bisex Queensland Fruit Fly (Bactrocera tryoni Froggatt) Release Programs
Source: Insects. 2022 Mar 9;13(3):269. doi: 10.3390/insects13030269 (PMC8952853; doi:10.3390/insects13030269)
Supplement: Supplementary file 1 [file insects-13-00269-s001.zip › insects-1555522-supplementary.pdf]

Supplementary Table S1. Mean number of stings and *Helicoverpa* spp. damage across orchards and dates of sampling: number of bins, average fruit/bin sampled, and fruit type (Pl = Plum, WP = White Peach, YP = Yellow Peach, Nc = Nectarine). and variety (QG:Queen Garnet, SA:Snow Angel, MP:May Princess, SP:Spring Princess, ZF:Zee Fire, BN:Black Nectar, CL:Crimson Lady, ET:Ebony Treat, PM:Purple Majesty).

Date	Bins	Av Fruit/bin	Fruit (var.): Number Fruit	*Proportion of B. tryoni* Stings in Fruit	*Proportion of Helicoverpa* spp. Larvae
			Traprock (6807 fruit in total) (SIT)		
2016-10-27	16	37.8	WP (SA):605	0.023	
2016-10-28	23	34.3	WP (SA):700, YP (MP):90	0.058	
2016-10-29	24	30	WP (SA):720	0.018	
2016-11-08	35	27.1	YP (MP):120, YP (SP):830	0.036	
2016-11-09	19	67.5	Nc (ZF):690, YP (MP):592	0.129	
2016-11-10	30	30	YP (SP):900	0.041	
2016-12-06	23	41.7	Pl (BN):270, YP (CL):690	0.075	
2016-12-08	5	120	Pl (BN):600	0.100	
			Top Lawson (4674 fruit in total)(C)		
2016-12-20	26	30	Pl (ET):780	0.018	0.023
2016-12-21	18	33	Pl (PM):594	0.017	0
2016-12-22	66	30	Pl (PM):1980	0.036	0.014
2016-12-23	44	30	Pl (PM):1320	0.024	0.009
			Warroo (8315 fruit in total)(SIT)		
2017-01-30	46	30	Pl (QG):1380	0.128	0.062
2017-01-31	46	35	Pl (QG):1610	0.147	0.155
2017-02-01	37	35	Pl (QG):1295	0.134	0.142
2017-02-07	42	35	Pl (QG):1470	0.073	0.209
2017-02-08	42	35	Pl (QG):1470	0.075	0.214
2017-02-09	28	38.9	Pl (QG):1090	0.089	0.219
